# Intra‐fractional motion error during HyperArc stereotactic radiosurgery on patients with brain metastases: Comparison of open and full‐face clamshell‐style immobilization devices

**DOI:** 10.1002/acm2.13536

**Published:** 2022-01-20

**Authors:** Shingo Ohira, Riho Komiyama, Naoyuki Kanayama, Yoshihiro Ueda, Shoki Inui, Masayoshi Miyazaki, Masahiko Koizumi, Koji Konishi

**Affiliations:** ^1^ Department of Radiation Oncology Osaka International Cancer Institute Osaka Japan; ^2^ Department of Medical Physics and Engineering Osaka University Graduate School of Medicine Suita Japan

## Abstract

**Purpose:**

To compare the intrafractional motion error (IME) during stereotactic irradiation (STI) in patients with brain metastases immobilized using open‐ (Encompass) and full‐face (DSPS) clamshell‐style immobilization devices.

**Methods:**

Encompass (38 patients) and DSPS (38 patients) were used for patient immobilization, and HyperArc plans with three to four non‐coplanar beams were generated to deliver 25 to 35 Gy in three to five fractions. Cone‐beam computed tomography (CBCT) was performed on patients before and after the treatment. Moreover, the difference in patient position between the two CBCT images was considered as the IME. The margins to compensate for IME were calculated using the van Herk margin formula.

**Results:**

For Encompass, the mean values of IME in the translational setup were 0.1, 0.2, and 0.0 mm in the anterior–posterior, superior–inferior, and left–right directions, respectively, and the mean values of IME about rotational axes were −0.1, 0.0, and 0.0° for the Pitch, Roll, and Yaw rotations, respectively. For DSPS, the mean values of IME in the translational setup were 0.2, 0.2, and 0.0 mm in the anterior–posterior, superior–inferior, and left–right directions, respectively, and the mean values of IME about rotational axes were −0.1, −0.1, and 0.0° for the Pitch, Roll, and Yaw rotations, respectively. No statistically significant difference was observed between the IME of the two immobilization systems except in the anterior–posterior direction (*p* = 0.02). Moreover, no statistically significant correlation was observed between three‐dimensional IME and treatment time. The margin compensation for IME was less than 1 mm for both immobilization devices.

**Conclusions:**

The IME during STI using open‐ and full‐face clamshell‐style immobilization devices is approximately equal considering the adequate accuracy in patient positioning.

## 1 INTRODUCTION

The occurrence of brain metastases increases owing to systemic therapy and advances in imaging modalities. Moreover, the management of brain metastases that deteriorates the patients’ quality of life is a major problem in modern radiotherapy.^1^ Linear accelerator‐based stereotactic irradiation (STI) is increasingly used in conjunction with volumetric images, a six‐degrees‐of‐freedom (6 DoF) couch, and non‐coplanar volumetric modulated arc therapy^2^, ^3^ to manage brain metastases. The advanced HyperArc radiation therapy can generate highly conformal doses to the target while minimizing doses to the surrounding organs at risk with a minimal workload.^4^

Large radiation doses are delivered in a small fraction (1 to 5 fractions) during the STI for patients with brain metastases, and a narrow margin (1–3 mm) is added to gross tumor volume (GTV) (or clinical target volume) to minimize radiation‐induced side effects, such as radionecrosis.^5^ Kirkpatrick et al demonstrated that a 3 mm margin posed a higher risk of radionecrosis than a 1 mm margin at comparable rates of local control.^6^ Thus, accurate patient positioning is imperative for the success of STI with a narrow margin. In modern radiotherapy, the interfractional motion error can be corrected using the 6DoF. However, minimizing the intrafractional motion error (IME) is still a challenging task.

A noninvasive individualized thermoplastic immobilization device is typically used to stabilize and maintain a patient's position during linear accelerator‐based STI. Ohtakara et al. demonstrated that a clamshell‐style immobilization device consisting of facial and occipital parts of a thermoplastic mask resulted in a smaller IME than the IME of a conventional four‐point thermoplastic mask and provided positional stability acceptable for the implementation of STI.^7^ None of the researchers have compared the IME during STI of the two relatively new commercially available clamshell‐style immobilization devices: the double‐shell positioning system (DSPS) (Macromedics, The Netherlands) and QFix Encompass SRS immobilization system (Avondale, PA, USA).

The aim of this study was to compare the IME during the STI for patients with brain metastases immobilized with open‐face (Encompass) and full‐face (DSPS) clamshell‐style immobilization devices as well as to calculate margins to compensate for the IME.

## 2 MATERIALS AND METHODS

### 2.1 Patients and simulation

**FIGURE 1:**
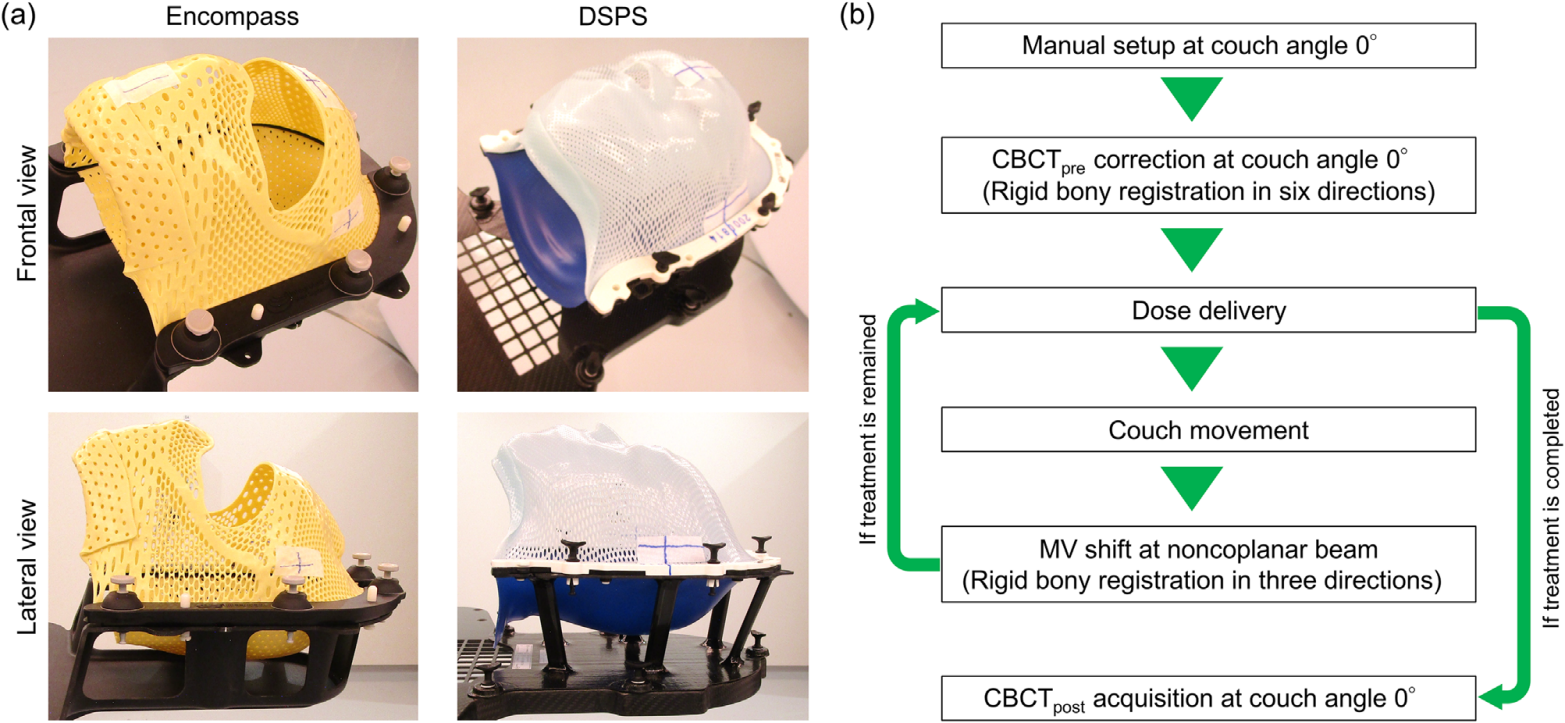
(a) Frontal and lateral views of the Encompass and DSPS immobilization systems. (b) Workflow of image acquisition (cone‐beam CT (CBCT) and megavoltage (MV) portal image) and patients’ position correction

This retrospective study including 76 patients who underwent fractionated STI was approved by Institutional Review Board. The written informed consent was waived because of the retrospective design. Table 1 lists the patient characteristics. We immobilized the patients (except when patients felt smothered) for simulation using clamshell‐style immobilization devices (38 patients using Encompass and 38 using DSPS) while wearing medical masks to avoid the risk of interpersonal infection between patients and medical staff.^8^ Encompass utilizes a rigid thermoplastic material that is 50% more rigid than standard thermoplastic, and the facial mask is open for patient comfort (Figure 1a). The DSPS consists of two thermoplastic masks: a thin and flat occipital mask that is rigid enough to hold the patient's head during molding, and a mesh fabric facial mask that covers the patient's entire face with a small hole in the nasal region (Figure 1 a). We used a dual‐energy computed tomography (CT) system (Revolution HD; GE Medical Systems, Milwaukee, WI) for imaging and the images were reconstructed with 1 mm slice thickness.

**TABLE 1:** Patient characteristics enrolled in this study

	**Encompass**	**DSPS**
Number of patients (*n*)	38	38
Male/female (*n*)	16/22	19/19
Age (*y*), median (range)	67 (30–83)	66 (37–85)
Number of metastases, median (range)	3 (1–25)	3 (1–33)
Treatment plan (*n*)		
Prescription dose (25/30/32/35 Gy)	1/8/1/28	0/9/0/29
Number of fractions (3/4/5 fractions)	7/1/30	8/0/30
Number of treatment fields (3/4 arcs)	1/37	2/36

### 2.2 Treatment planning and dose delivery

The CT images were loaded into a treatment planning system (Eclipse; Varian Medical Systems, Palo Alto, CA, USA). A radiation oncologist delineated a GTV and added a margin of 1–2 mm to generate the planning target volume (PTV). HyperArc plans^4^ were generated using three to four non‐coplanar beam arrangements (couch angle of 0°, 45° (and/or 315°), 90° (or 270°) with automatic couch movement to deliver 25–35 Gy to cover 95% of the PTV in three to five fractions for all the patients (Table 1). We used treatment units of TrueBeam STX and Edge equipped with a 2.5 mm wide multileaf collimator and a 6 MV photon beam (flattening filter‐free) with a dose rate of 1400 monitor units per minute for treating patients.

We immobilized the patients and obtained 1 mm slices of cone‐beam CT images (CBCT_pre_) for their treatment. The CBCT and CT images acquired in the simulation were automatically registered using a six‐dimensional rigid bony registration (anterior–posterior, AP; superior–inferior, SI; left–right, LR; pitch; roll; yaw), and the doses were delivered after patients’ position correction (Figure 1b). Furthermore, we acquired megavoltage (MV) portal images (two or three times) at the couch angle of 45° (and/or 315°), 90° (or 270°), and the patients’ position was corrected by using the MV images and the corresponding reconstructed radiographs with bony registration. The couch was shifted in three directions (SI, LR, and yaw) because the MV images were acquired using the anterior or posterior beam (gantry angle of 0° or 180°).^9^ We acquired CBCT again to assess the IME during STI dose delivery (CBCT_post_). The time between acquisitions of CBCT_pre_ and CBCT_post_ was the treatment time. During treatment, the surface‐guided patient setup was not performed for both immobilization devices.

### 2.3 Data analysis

The magnitude of couch shift for patients’ position correction using MV images between Encompass and DSPS group was compared in the three directions. The positional displacement of patients between the CBCT_pre_ and CBCT_post_ in the six directions was determined as the IME during the STI dose delivery (IME_AP_, IME_SI_, IME_LR_, IME_Pitch_, IME_Roll_, and IME_Yaw_). The three‐dimensional (3D) translational IME was given by $\sqrt{IME_{AP}^{2}+IME_{SI}^{2}+IME_{LR}^{2}}$, and the 3D rotational IME was given by $\sqrt{IME_{Pitch}^{2}+IME_{Roll}^{2}+IME_{Yaw}^{2}}$. Mann–Whitney *U* test was performed to determine the statistical differences between the couch shift and IME in the Encompass and DSPS immobilization systems. The absolute value of Spearman rank correlation coefficient for the 3D translational/rotational IME and treatment time data sets was considered “weak,” “moderate,” and “strong” when 0 ≤ *r_s _* < 0.4, 0.4 ≤ *r_s _* < 0.6, and 0.6 ≤ *r_s_*. All statistical analyses were performed using SPSS software (version 27; IBM, Armonk, NY, USA), and statistical significance was set at *p *< 0.05.

The systematic and random errors were the mean and standard deviation (SD) of IME, respectively, through the course of the treatment course of each patient. The values of Σ and σ determined the SD of the systematic errors for all patients and the root mean square of the random error. Finally, the margin (M) to compensate for IME in the three directions (AP, SI, and LR) was calculated using the formula presented by van Herk et al.^10^: M = 2.5Σ + 0.7σ.

## 3 RESULTS

**FIGURE 2:**
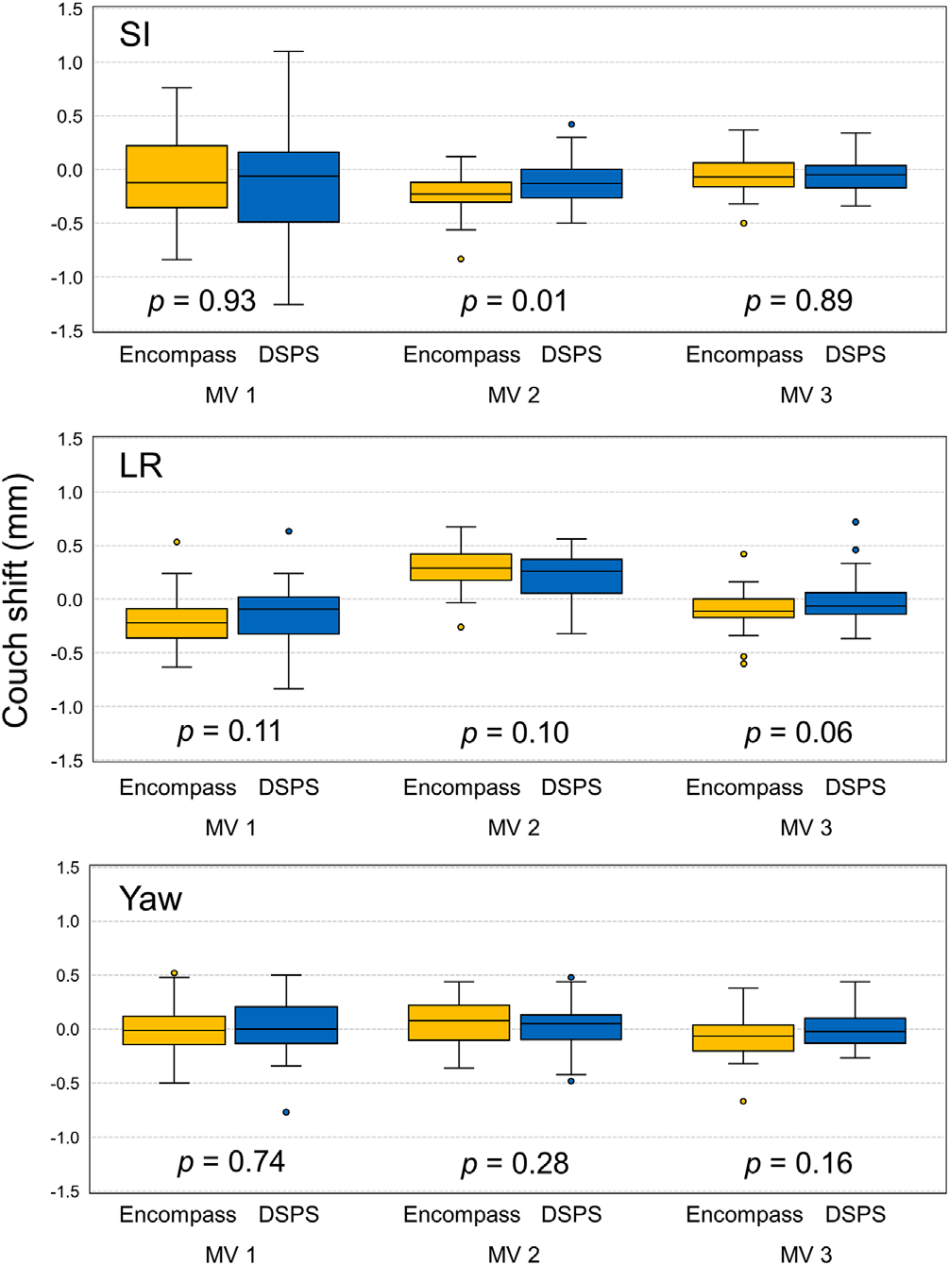
Magnitude of the couch shift for patients’ position correction using megavoltage (MV) portal images acquired at the couch angle of 45° (and/or 315°), 90° (or 270°) in the Encompass and DSPS groups. The MV images were acquired with using the anterior or posterior beam (gantry angle of 0° or 180°)

We analyzed 175 and 174 treatment sessions (520 and 514 MV images) in the Encompass and DSPS groups, respectively, and Figure 2 shows the couch shift for patients’ position correction using MV images in three directions. The comparable means of the couch shift were obtained in any directions between Encompass (≤0.3 mm) and DSPS (≤0.2 mm). The statistically significant difference was observed only in couch shift using MV image in the SI direction (*p* = 0.01).

**FIGURE 3:**
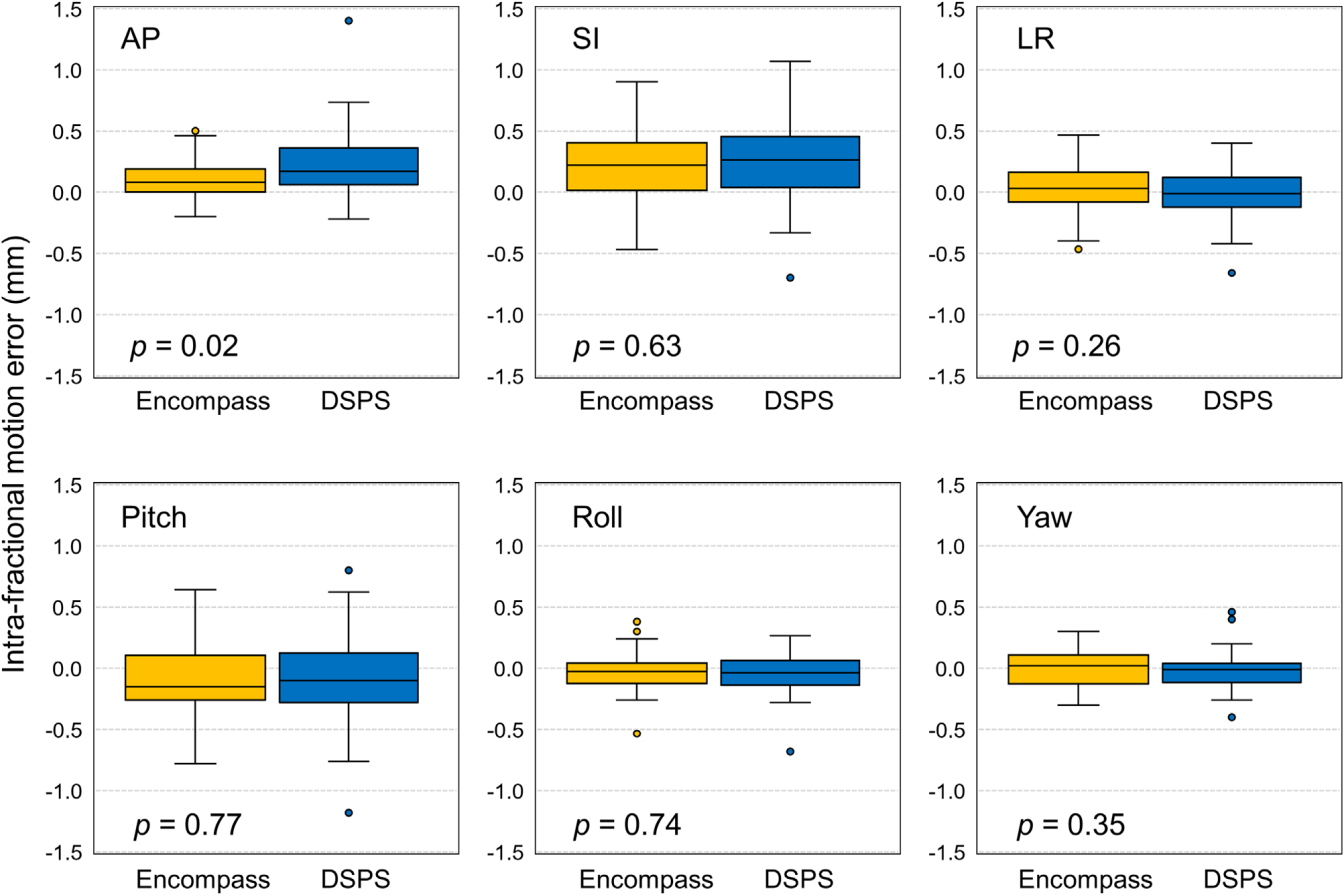
Comparison of the intra‐fractional motion error (IME) in the six directions between the Encompass and DSPS immobilization devices. The IME was determined as the difference in the patient position between the cone‐beam CT acquired before and after dose delivery

Figure 3 presents a comparison of the IME in the six directions between Encompass and DSPS. For Encompass, the means of IME were equal to 0.1, 0.2, and 0.0 mm in the AP, SI, and LR directions, respectively, and –0.1, 0.0, and 0.0° in the Pitch, Roll, and Yaw directions. For DSPS, the means of IME were equal to 0.2, 0.2, and 0.0 mm in the AP, SI, and LR directions, and −0.1, −0.1, and 0.0° in the Pitch, Roll, and Yaw directions. We observed no statistically significant difference in the IME between the two immobilization systems except in the AP direction (*p* = 0.02).

**FIGURE 4:**
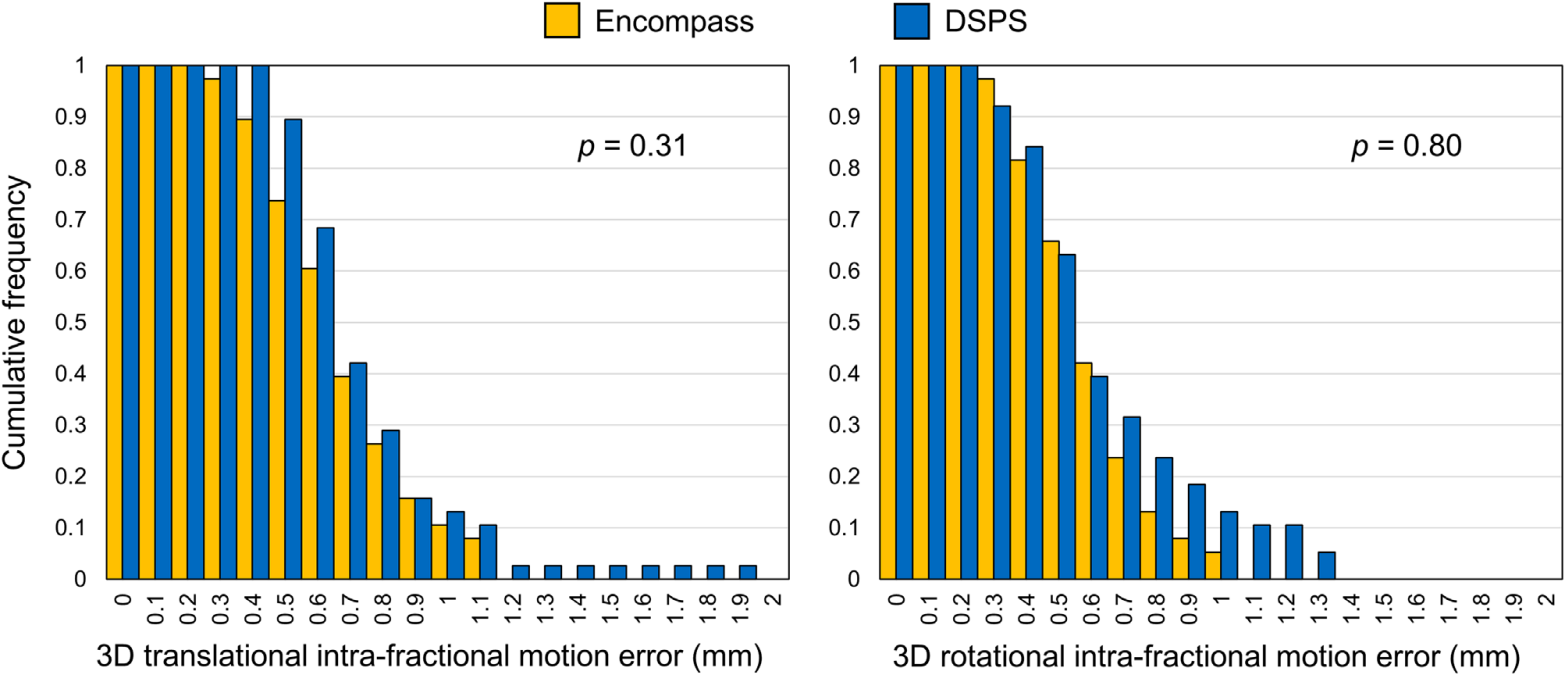
Cumulative frequency of the three‐dimensional (3D) translational and rotational intra‐fractional motion error for the Encompass and DSPS immobilization devices

**FIGURE 5:**
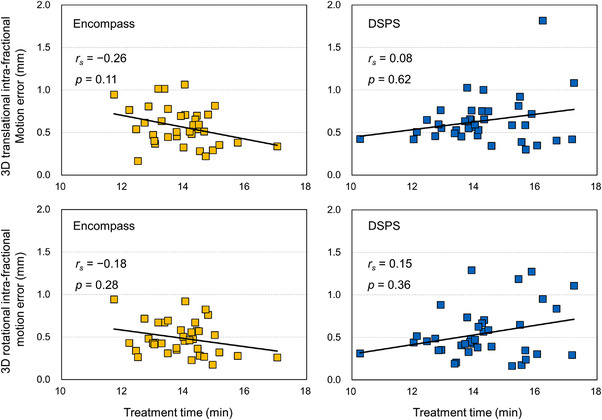
Correlation between the three‐dimensional (3D) translational/rotational intra‐fractional motion error and treatment time for the Encompass and DSPS immobilization devices. The absolute value of Spearman rank correlation coefficient was considered “weak,” “moderate,” and “strong” when 0 ≤ *r_s  _* <0.4, 0.4 ≤ *r_s _* <0.6, and 0.6 ≤ *r_s_*

The cumulative frequencies of the 3D translational and rotational IME are shown in Figure 4. The maximum 3D translational and rotational IME were 1.1 mm and 1° in the Encompass immobilization system, respectively, and the maximum 3D translational and rotational IME were 1.9 mm and 1.3° in the DSPS immobilization system. We did not observe any statistically significant difference in the 3D translational (0.6 ± 0.2 mm and 0.6 ± 0.3 mm for Encompass and DSPS, respectively, *p* = 0.31) and rotational (0.5° ± 0.2 mm and 0.5 ± 0.3 mm for Encompass and DSPS, respectively, *p* = 0.80) between the two immobilization devices. Figure 5 demonstrates the correlation between the 3D translational/rotational IME and treatment time. We observed a weak negative correlation (*p* > 0.05) between the 3D translational (*r_s_* = ‐0.26) and rotational (*r_s_* = ‐0.18) IME for Encompass, and a weak positive correlation (*p* > 0.05) between the 3D translational (*r_s_* = 0.08) and rotational (*r_s_* = 0.15) IME for DSPS.

The margins calculated to compensate for the IME using van Herk's formula during STI dose delivery are listed in Table 2. We obtained comparable Σ between the Encompass (0.2, 0.3, and 0.2 mm in the AP, SI, and LR directions, respectively) and DSPS (0.3, 0.3, and 0.2 mm in the AP, SI, and LR directions, respectively) cases. Moreover, σ in the AP (0.2 and 0.2 mm), SI (0.3 and 0.2 mm), and LR (0.2 and 0.3 mm) directions were approximately equal between the two immobilization devices. Finally, a margin of ≤1 mm was achieved in each direction for both immobilization devices.

**TABLE 2:** Margins to compensate for the intra‐fractional motion error in the translational directions. The margin was calculated using the formula presented by van Herk et al.^10^: Margin = 2.5Σ + 0.7σ

	**Encompass**			**DSPS**		
	**AP**	**SI**	**LR**	**AP**	**SI**	**LR**
Σ (mm)	0.2	0.3	0.2	0.3	0.3	0.2
σ (mm)	0.2	0.3	0.2	0.2	0.2	0.3
M (mm)	0.6	0.9	0.7	0.9	1.0	0.7

## 4 DISCUSSIONS

We compared the IME during STI dose delivery using open‐ (Encompass) and full‐face (DSPS) clamshell‐style immobilization devices. The STI based on the linear accelerator required a longer treatment time compared to conventional radiotherapy (2 Gy per fraction) because of the larger monitor unit, non‐coplanar beam arrangement, and 3D volumetric image registration. A comfortable immobilization device is ideal for STI. However, the accuracy of patient positioning cannot be compromised owing to the steep dose gradient for small targets with small margins. The dose gradient is steeper for HyperArc treatment than the dose gradient for conventional volumetric modulated arc therapy to minimize the doses to brain tissues.^4^ Thus, selecting the appropriate immobilization device is imperative for the success of STI treatment.

Numerous immobilization systems are available commercially for STIs (Table 3). Lewis et al. observed that the 3D IME between the biplanar X‐ray image registrations using a clamshell‐style immobilization system (model 41100; BrainLAB A.G., Heimstetten, Germany) was less than 0.8 mm.^11^ Barnes et al. demonstrated that the 3D translational IME was well within 0.7 mm during treatment using Klarity thermoplastic masks (Klarity Medical Products, OH, USA) in conjunction with the Brainlab frameless stereotactic fixation system.^12^ Tryggestad et al. compared the immobilization accuracy of four types of thermoplastic immobilization systems (Civco, Kalona, IA), and observed that the 3D IME was approximately equal to 1 mm for all the immobilization systems.^13^ We compared the IME for two relatively new immobilization devices (Encompass and DSPS) during HyperArc treatment and observed that the mean 3D IME was less than 1 mm and 1° for both the devices. Therefore, a 1 mm margin was adequate to compensate for the IME during STI dose delivery for patient position correction.

**TABLE 3:** Summary of 3D translational intra‐fractional motion error (IME) with noninvasive thermoplastic immobilization device

**References**	**Immobilization device**	**Number of patients**	**3D translational IME (mm)**
Lewis et al. [11]	Brainlab thermoplastic (head)	104	0.8 ± 0.5
Barnes et al. [12]	Klarity thermoplastic (head)	101	well within 0.7
**Tryggestad et al. [13]**	Civco Type‐S thermoplastic (head)	20	1.1 ± 1.2
	Civco Uni‐Frame thermoplastic (head)	9	1.1 ± 1.1
	Civco Type‐S thermoplastic (head and shoulder)	81	0.7 ± 0.9
	Civco Type‐S thermoplastic (head and shoulder) + bite block	11	0.7 ± 0.8
**Present study**	Encompass thermoplastic (head)	38	0.6 ± 0.2
	DSPS thermoplastic (head)	38	0.6 ± 0.3

The most common PTV margin in clinical practice was equal to 2 mm in the survey of the Japanese Radiation Oncology Study Group.^5^ A wide margin can guarantee the dosage delivery to the tumor. However, this can increase the radiation doses to the surrounding normal tissues. Kirkpatrick et al. demonstrated that the minimum dose to PTV and the volume of the brain tissue receiving 12 Gy dosage were significantly higher in the treatment plans with a 3 mm margin than in the treatment plans with a 1 mm margin.^6^ The local control after STI was comparable for both treatment plans. However, the risk of radionecrosis was significantly higher in the treatment plans with a 3 mm margin than in the treatment plans with a 1 mm margin. IME can be a dominant factor in the PTV margin calculation because the geometric uncertainty (e.g., radiation isocenter and couch positioning accuracy) in the modern linear accelerator is limited.^14^

The management of IME is an important area of research in radiotherapy. We used the megavoltage portal image to correct the patient position to minimize the IME and observed no time trend for both open‐ and full‐face immobilization devices. The methodology using a megavoltage portal image required a long time to acquire images and analyze patient position correction. This hampered the fully automated dose delivery and couch movement with Hyperarc. For an open‐face immobilization system, the surface‐guided patient setup can manage real‐time IME with short‐time patient position correction. Lee et al. demonstrated that the surface‐guided patient setup provided patient position correction in 0.8 min whereas the CBCT and 2D X‐ray image provided patient position correction in 3.4 and 1.1 min, respectively. Therefore, the surface‐guided patient setup can efficiently manage IME and accelerate the radiotherapy treatment.^15^

The limitations of our research are as follows: (1) the number of patients enrolled in our research was limited, and further research is needed to calculate margins to compensate for a variety of patient characteristics, including severe performance status. (2) Patient correction using the megavoltage portal image was limited to three directions (SI, LR, and Yaw), and CBCT correction was required during dose delivery to reduce the IME in the other directions (AP, Pitch, and Roll). (3) the mouthpiece was not used for patient immobilization in this study. Tomihara et al. demonstrated the DSPS combined with a mouthpiece achieved a smaller IME than the IME without a mouthpiece.^16^ (4) The margins calculated in our research do not account for uncertainties, such as the rotational IME, geometric uncertainty of the linear accelerator, and interobserver variability of the target delineation. An additional margin may be needed to compensate for these uncertainties. Despite these limitations, the quantitative data obtained from our research provide useful information for selecting appropriate immobilization devices in the STI for brain metastases.

The accuracy of patient positioning using open (Encompass) and full‐face (DSPS) clamshell‐style immobilization devices was approximately equal during the STI, and the required margins were less than 1 mm. Moreover, no significant correlation was observed between the 3D IME and treatment time for either immobilization device.

## CONFLICT OF INTEREST

The authors have no conflicts of interest to declare in relation to this study.

## PRESENTATION AT A CONFERENCE

None.

## FUNDING INFORMATION

This study was supported by JSPS KAKENHI Grant (Grant‐in‐Aid for Scientific Research (C) 21K07742).

## AUTHORS' CONTRIBUTIONS

Concept and design: OS, KR, YU, IS. Data analysis: OS, KR. Manuscript preparation: all authors. All authors read and approved the final manuscript.

## Supporting information

Supplementary information
