# Supplementary material for: Intra‐fractional motion error during HyperArc stereotactic radiosurgery on patients with brain metastases: Comparison of open and full‐face clamshell‐style immobilization devices
Source: J Appl Clin Med Phys. 2022 Jan 20;23(4):e13536. doi: 10.1002/acm2.13536 (PMC8992945; doi:10.1002/acm2.13536)
Supplement: Supplementary file 1 — Supplementary information [file ACM2-23-e13536-s001.docx]

**Title**

Intra-fractional motion error during HyperArc stereotactic radiosurgery on patients with brain metastases: Comparison of open and full-face clamshell-style immobilization devices.

**Short running title**

Comparison of intra-fractional motion

**Authors**

Shingo Ohira ^1,2^, Riho Komiyama ^1^, Naoyuki Kanayama ^1^, Yoshihiro Ueda ^1^, Shoki Inui ^1^, Masayoshi Miyazaki ^1^, Masahiko Koizumi ^2^, Koji Konishi ^1^.

^1^Department of Radiation Oncology, Osaka International Cancer Institute, Osaka, Japan.

^2^Department of Medical Physics and Engineering, Osaka University Graduate School of Medicine, Suita, Japan.

**Details of corresponding author**

Shingo, Ohira, Ph.D.,

Department of Radiation Oncology, Osaka International Cancer Institute, 3-1-69 Otemae, Chuo-ku, Osaka, 537-8567 Osaka, Japan.

Tel: +81-6-6945-1181, Fax: +81-6-6945-1900

e-mail: [oohira-si@mc.pref.osaka.jp](mailto:oohira-si@mc.pref.osaka.jp)

**Conflicts of Interest:** The Authors have no conflicts of interest to declare in relation to this study.

**Presentation at a conference:** None.

**Funding**

This study was supported by JSPS KAKENHI Grant (Grant-in-Aid for Scientific Research (C) 21K07742).

**Authors' contributions**

Concept and design: OS, KR, YU, IS. Data analysis: OS, KR. Manuscript preparation: all authors. All authors read and approved the final manuscript.
